# Implications of historical height loss for prevalent vertebral fracture, spinal osteoarthritis, and gastroesophageal reflux disease

**DOI:** 10.1038/s41598-020-76074-6

**Published:** 2020-11-04

**Authors:** Masaki Nakano, Yukio Nakamura, Takako Suzuki, Tsukasa Kobayashi, Jun Takahashi, Masataka Shiraki

**Affiliations:** 1 Department of Orthopaedic Surgery, Shinshu University School of Medicine, 3-1-1 Asahi, Matsumoto, Nagano, 390-8621 Japan; 2 Department of Human Nutrition, Faculty of Human Nutrition, Tokyo Kasei Gakuin University, 22 Sanban-cho, Chiyoda-ku, Tokyo, 102-8341 Japan; 3 Research Institute and Practice for Involutional Diseases, 1610-1 Meisei, Misato, Azumino, Nagano 399-8101 Japan

**Keywords:** Diseases, Endocrinology

## Abstract

We recently uncovered an association between spinal osteoarthritis and height loss that was independent of incident vertebral fracture. However, the optimal cut-off value of historical height loss (HHL) for discriminating spinal osteoarthritis has not been reported. This cross-sectional study aimed to evaluate the implications of HHL for prevalent vertebral fracture, spinal osteoarthritis, and other co-morbidities in postmenopausal women from the Nagano Cohort Study. In total, 942 Japanese postmenopausal outpatients (mean age: 66.7 years) were investigated. HHL was estimated by arm span – body height difference. Multiple logistic regression analysis revealed significant independent associations of HHL with prevalent vertebral fracture (odds ratio [OR] 1.89; 95% confidence interval [CI] 1.55–2.29), spinal osteoarthritis (OR 1.57; 95% CI 1.31–1.88), and gastroesophageal reflux disease (GERD) (OR 1.75; 95% CI 1.34–2.28) after adjustment for other confounders. Receiver operating characteristic curve analysis of HHL was conducted to discriminate the prevalence of co-morbidities. The optimal cut-off value as defined by the Youden index for prevalent vertebral fracture, spinal osteoarthritis, and GERD was 4.95 cm (area under the curve [AUC] 0.740; 95% CI 0.704–0.776), 2.75 cm (AUC 0.701; 95% CI 0.667–0.735), and 5.35 cm (AUC 0.692; 95% CI 0.629–0.754), respectively. Better understanding of the above relationships and proposed cut-off values will be useful for improving the diagnosis, care management, and quality of life in elderly patients.

## Introduction

Height loss with advancing age is a common process that is related to intervertebral disc degeneration and vertebral compression fracture^1,2^. Historical height loss (HHL) has been associated with a variety of negative consequences, including low back pain, respiratory disease, hypertension, and cerebro/cardiovascular events^3–5^. Moreover, a relationship between HHL and gastroesophageal reflux disease (GERD), a disorder within the chest cavity as well as respiratory disease, was reported in Japanese elderly individuals^6^. The impact of height loss on mortality rate has been documented as well^5,7^.

The association between vertebral fracture and HHL has been well investigated. Siminoski et al.^8^ and Yoh et al.^9^ proposed cut-off values of HHL for discriminating prevalent vertebral fracture to be 6.0 cm and 4.0 cm, respectively. As another major vertebral disorder with aging, spinal osteoarthritis is characterized as a degenerative joint disease that specifically affects the facet joints and cartilage or ligaments of the vertebral column. We recently uncovered that the prevalence of spinal osteoarthritis was associated with height loss rate independently of incident vertebral fracture in a longitudinal study conducted on postmenopausal women from the Nagano Cohort Study^10^. However, the optimal cut-off value of HHL for detecting spinal osteoarthritis has not yet been addressed.

The present cross-sectional study aimed to evaluate the implications of HHL for the prevalence rate of vertebral fracture, spinal osteoarthritis, and other co-morbidities such as diabetes mellitus (DM), dyslipidemia, hypertension, vascular events, and GERD in Japanese postmenopausal women enrolled in the Nagano Cohort Study. Significant associations of HHL with prevalent vertebral fracture, spinal osteoarthritis, and GERD prevalence were further analyzed to calculate the optimal HHL cut-off values for detecting those disorders.

## Methods

The study protocol of this investigation was reviewed by the ethics committee of the Research Institute and Practice for Involutional Diseases, Japan, prior to commencement and was conducted in accordance with the principles of the Declaration of Helsinki. Comprehensive written informed consent was provided from all participants.

### Study subjects

Started in 1993, the Nagano Cohort Study contains the medical records of postmenopausal outpatients treated at a primary care institute in Nagano Prefecture, Japan^10,11^. Patients with a critical illness (e.g., terminal cancer), acute illness (e.g., pneumonia or acute vascular disease), or secondary osteoporosis (e.g., steroid use or primary hyperparathyroidism) were excluded from the study. Subjects who could not walk without assistance were also excluded. In total, 942 Japanese postmenopausal patients (mean age: 66.7 years) enrolled during the course of the Nagano Cohort Study were investigated in this study.

### Data collection for patient characteristics

The body height and weight of patients were evaluated by standard procedures for calculations of body mass index (BMI, kg/m^2^). Arm span was measured to estimate the patient's tallest height^12,13^. We used dual energy X-ray absorptiometry (DXA; PRODIGY, GE Healthcare Lunar, Madison, WI) to determine bone mineral density (BMD) of the lumbar spine and total hip. Serum levels of albumin, hemoglobin A_1c_, total cholesterol, and triglycerides were also measured.

The prevalence of co-morbidities, including DM, dyslipidemia, hypertension, vascular events, and GERD, was determined by the diagnostic criteria of each disease and/or relevant treatment records as we described previously^14^. The diagnostic criteria for these co-morbidities are described as follows: DM was diagnosed if hemoglobin A_1c_ level was ≥ 6.5% or when patients were actively treated for DM. Dyslipidemia was defined as low-density lipoprotein-cholesterol ≥ 140 mg/dL, high-density lipoprotein-cholesterol < 40 mg/dL, or postprandial triglycerides ≥ 200 mg/dL. Here, we measured serum triglycerides levels in a postprandial state because the levels of triglycerides in a fasting and postprandial state have reportedly been highly correlated^15^. Hypertension was diagnosed when systolic blood pressure was persistently > 140 mmHg, diastolic pressure was persistently > 90 mmHg, or anti-hypertensive drugs were used. The prevalence of vascular events was defined as having clinically diagnosed cerebro-vascular or ischemic heart disease. GERD prevalence was discriminated by endoscopic screening for esophagitis.

### Prevalent vertebral fracture and spinal osteoarthritis diagnosis

Prevalent vertebral fracture was diagnosed by semi-quantitative analysis of a baseline X-ray film of the thoracic and lumbar spine (T4–L4)^16^. Osteoarthritis of the spine was detected by X-ray film observation of the T4–L4 vertebrae. The degree of spinal degeneration was assessed in accordance with the Kellgren–Lawrence (KL) grading system^17^, whereby KL grade ≥ 2 was judged as spinal osteoarthritis.

### Statistical analyses

Numerical data are presented as the mean ± standard deviation. The number and proportion of patients with prevalent vertebral fracture, spinal osteoarthritis, and other co-morbidities were recorded as well. HHL was estimated as the difference between the recorded arm span and body height of each patient^4,6,12,13^. We conducted quartile analysis for the prevalence rate of vertebral fracture, spinal osteoarthritis, and co-morbidities based on estimated HHL results. The significance of differences in each analysis was evaluated by the chi-squared test.

Multiple logistic regression analysis was performed to investigate the impact of HHL on the prevalence of vertebral fracture, spinal osteoarthritis, and co-morbidities. The analysis model was adjusted for the confounders of age and BMI. In addition, receiver operating characteristic (ROC) curves for HHL were constructed to discriminate prevalent vertebral fracture, spinal osteoarthritis, and GERD prevalence. The area under the ROC curve (AUC) with 95% confidence interval (CI) was calculated, and the optimal cut-off value was defined by the Youden index (maximum of [sensitivity + specificity − 1]).

A two-tailed *P*-value of < 0.05 was considered to be statistically significant in all analyses. All statistical tests were conducted using R version 3.6.0 software^18^.

## Results

### Characteristics of study subjects

A total of 942 participants met the selection criteria of the present study. All patients were postmenopausal and mean age was 66.7 years. The characteristics of the study subjects are summarized in Table 1. Patients with DM, dyslipidemia, hypertension, vascular events, and GERD were present in 14.2% (*n* = 134), 48.9% (*n* = 461), 56.3% (*n* = 530), 14.6% (*n* = 138), and 8.5% (*n* = 80) of the study population, respectively. The respective number of patients diagnosed as having prevalent vertebral fracture and spinal osteoarthritis (i.e., KL grade ≥ 2) was 265 (28.1%) and 614 (65.2%).

**Table 1:** Characteristics of study subjects.

	No	Mean ± SD	Min	Med	Max
Age, years	942	66.7 ± 9.9	42	66	93
Body height, cm	942	151.1 ± 6.2	131.0	151.0	173.0
Body weight, kg	942	51.5 ± 8.3	27.5	51.0	84.0
BMI, kg/m^2^	942	22.5 ± 3.3	14.3	22.1	38.3
Arm span, cm	942	152.6 ± 6.5	130.0	153.0	175.5
Lumbar BMD, g/cm^2^	942	0.94 ± 0.20	0.45	0.92	1.72
Hip BMD, g/cm^2^	909	0.76 ± 0.14	0.32	0.76	1.21
Albumin, g/dL	862	4.2 ± 0.3	3.1	4.2	5.3
HbA1c, %	906	5.4 ± 0.7	3.5	5.3	10.6
Total cholesterol, mg/dL	941	205.0 ± 35.4	92.0	204.0	397.0
Triglycerides*, mg/dL	941	143.6 ± 82.9	25.0	122.0	718.0

*Triglycerides were measured with the subject in a postprandial state.

SD, standard deviation; Min, minimum; Med, median; Max, maximum; BMI, body mass index; BMD, bone mineral density; HbA1c, hemoglobin A_1c_.

### Impact of HHL on co-morbidity prevalence

Quartile analysis based on estimated patient's HHL for the prevalence rate of vertebral fracture, spinal osteoarthritis, and other co-morbidities including DM, dyslipidemia, hypertension, vascular events, and GERD revealed that higher HHL quartiles (i.e., patients with more profound height loss) exhibited a significantly higher prevalence of vertebral fracture, spinal osteoarthritis, hypertension, vascular events, and GERD (Table 2). The range of each quartile is presented in the footnotes of Table 2.

**Table 2:** Number of patients with each co-morbidity by quartile of HHL (arm span − body height).

	Quartile 1	Quartile 2	Quartile 3	Quartile 4	*P*-value
Prevalent vertebral fracture, No. (%)	27 (11.5)	36 (15.3)	70 (29.7)	132 (56.2)	< 0.001
Spinal osteoarthritis, No. (%)	105 (44.7)	139 (58.9)	165 (69.9)	205 (87.2)	< 0.001
Diabetes mellitus, No. (%)	33 (14.0)	45 (19.1)	30 (12.7)	26 (11.1)	0.08
Dyslipidemia, No. (%)	125 (53.2)	128 (54.2)	108 (45.8)	100 (42.6)	0.03
Hypertension, No. (%)	99 (42.1)	115 (48.7)	144 (61.0)	172 (73.2)	< 0.001
Vascular events, No. (%)	16 (6.8)	30 (12.7)	39 (16.5)	53 (22.6)	< 0.001
GERD, No. (%)	9 (3.8)	13 (5.5)	22 (9.3)	36 (15.3)	< 0.001

Quartile 1 ranged from − 12.5 to − 0.2 cm (*n* = 235).

Quartile 2 ranged from − 0.1 to 3.7 cm (*n* = 236).

Quartile 3 ranged from 3.8 to 7.6 cm (*n* = 236).

Quartile 4 ranged from 7.7 to 24.0 cm (*n* = 235).

HHL, historical height loss; GERD, gastroesophageal reflux disease.

To evaluate for associations between HHL and the prevalence rates of vertebral fracture, spinal osteoarthritis, and co-morbidities, multiple logistic regression analysis was performed with adjustment for the confounders of age and BMI. In this analysis, prevalence of vertebral fracture, spinal osteoarthritis, hypertension, vascular events, and GERD showed statistically significant relationships with patient age, while spinal osteoarthritis, DM, dyslipidemia, hypertension, and vascular events prevalence were significantly associated with BMI (Table 3). On the other hand, the analysis model demonstrated the statistically significant independence of HHL as a determinant for prevalent vertebral fracture (odds ratio [OR] 1.89; 95% CI 1.55–2.29), spinal osteoarthritis (OR 1.57; 95% CI 1.31–1.88), and GERD (OR 1.75; 95% CI 1.34–2.28). However, HHL was not an independent determinant for the prevalence of hypertension and vascular events as well as DM and dyslipidemia in this model (Table 3).

**Table 3:** Multiple logistic regression analysis for prevalence of each co-morbidity by HHL (arm span − body height) with adjustment for patient age and BMI.

	Odds ratio	95% CI	*P*-value
**for Prevalent vertebral fracture**			
Age (years, + 1SD)	2.28	1.86–2.80	< 0.001
BMI (kg/m^2^, + 1SD)	1.13	0.96–1.32	0.16
HHL (cm, + 1SD)	1.89	1.55–2.29	< 0.001
**for Spinal osteoarthritis**			
Age (years, + 1SD)	2.05	1.72–2.46	< 0.001
BMI (kg/m^2^, + 1SD)	1.65	1.40–1.95	< 0.001
HHL (cm, + 1SD)	1.57	1.31–1.88	< 0.001
**for Diabetes mellitus**			
Age (years, + 1SD)	1.18	0.94–1.47	0.15
BMI (kg/m^2^, + 1SD)	1.74	1.46–2.08	< 0.001
HHL (cm, + 1SD)	0.82	0.66–1.03	0.09
**for Dyslipidemia**			
Age (years, + 1SD)	0.94	0.81–1.09	0.42
BMI (kg/m^2^, + 1SD)	1.43	1.25–1.64	< 0.001
HHL (cm, + 1SD)	0.87	0.75–1.01	0.06
**for Hypertension**			
Age (years, + 1SD)	2.02	1.70–2.40	< 0.001
BMI (kg/m^2^, + 1SD)	1.89	1.61–2.21	< 0.001
HHL (cm, + 1SD)	1.16	0.99–1.37	0.07
**for Vascular events**			
Age (years, + 1SD)	2.13	1.67–2.72	< 0.001
BMI (kg/m^2^, + 1SD)	1.44	1.20–1.72	< 0.001
HHL (cm, + 1SD)	1.13	0.91–1.40	0.26
**for GERD**			
Age (years, + 1SD)	1.39	1.05–1.85	< 0.05
BMI (kg/m^2^, + 1SD)	1.05	0.83–1.32	0.71
HHL (cm, + 1SD)	1.75	1.34–2.28	< 0.001

HHL, historical height loss; BMI, body mass index; SD, standard deviation; CI, confidence interval; GERD, gastroesophageal reflux disease.

### ROC curve analysis of HHL

**Figure 1:**
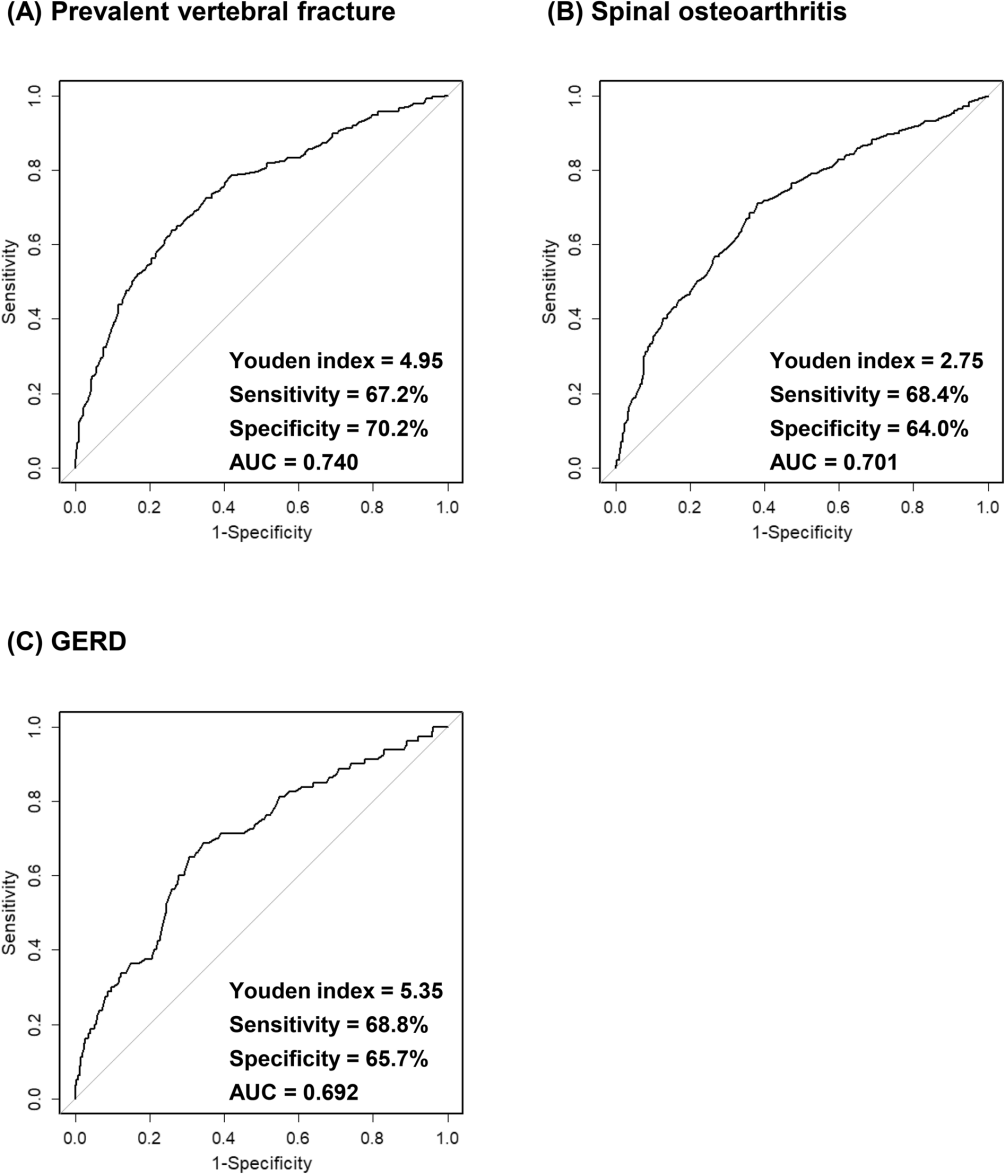
Receiver operating characteristic curves of historical height loss to discriminate the (**A**) prevalent vertebral fracture, (**B**) spinal osteoarthritis, and (**C**) GERD prevalence. The analysis was performed by using R version 3.6.0 software^18^. GERD, gastroesophageal reflux disease; AUC, area under the receiver operating characteristic curve.

ROC curves were plotted to identify HHL discrimination thresholds for prevalent vertebral fracture, spinal osteoarthritis, and GERD. The optimal cut-off value as defined by the Youden index for prevalent vertebral fracture, spinal osteoarthritis, and GERD was 4.95 cm (AUC 0.740; 95% CI 0.704–0.776), 2.75 cm (AUC 0.701; 95% CI 0.667–0.735), and 5.35 cm (AUC 0.692; 95% CI 0.629–0.754), respectively (Fig. 1). The optimal cut-off and AUC values of HHL for detecting the individual and combined prevalence of these disorders are presented in Supplementary Table 1.

### GERD prevalence and associated vertebral disorders

As shown in Table 4, patients with GERD exhibited a significantly higher presence of prevalent vertebral fracture and spinal osteoarthritis. The proportion of patients with prevalent vertebral fracture and spinal osteoarthritis in GERD patients was 45.0% (*P* < 0.001 vs. non-GERD patients [26.6%]) and 80.0% (*P* = 0.005 vs. non-GERD patients [63.8%]), respectively.

**Table 4:** Patients with GERD exhibited a significantly higher presence of vertebral disorders.

	GERD		*P*-value
	Negative (862 subjects)	Positive (80 subjects)	
Prevalent vertebral fracture, Positive, No. (%)	229 (26.6)	36 (45.0)	< 0.001
Spinal osteoarthritis, Positive, No. (%)	550 (63.8)	64 (80.0)	0.005

GERD, gastroesophageal reflux disease.

## Discussion

The present study demonstrated a significant implication of HHL on prevalent vertebral fracture, spinal osteoarthritis, and the co-morbidity of GERD. These findings were supported by significant multivariate analysis results and ROC curve-based cut-off HHL values for predicting each disorder. Furthermore, significant associations of GERD prevalence with prevalent vertebral fracture and spinal osteoarthritis indicated a possible new diagnostic application.

To date, vertebral compression fracture has been implicated as the most common cause of height loss, whereupon 4.0–6.0 cm of HHL was suggested as a cut-off value for detecting prevalent vertebral fracture^8,9^. In this study, we observed a significant association of HHL with prevalent vertebral fracture in multiple logistic regression analysis. The ROC curve model showed the optimal cut-off value of HHL to discriminate prevalent vertebral fracture to be 4.95 cm. These results were consistent with previous reports. In addition, we very recently reported on the association between spinal osteoarthritis, another major vertebral disorder with age, and height loss in a longitudinal study of postmenopausal women^10^. HHL was an independent predictor of the prevalence of spinal osteoarthritis in the current study, with an optimal cut-off value as defined by the Youden index of 2.75 cm. To the best of our knowledge, this study is the first to propose a value for detecting spinal osteoarthritis prevalence by HHL.

A previous report by Eguchi et al.^6^ demonstrated a relationship between HHL and an esophageal disorder, GERD. Their study included approximately 300 Japanese elderly men and women assessed using a questionnaire termed the Frequency Scale for the Symptoms of GERD (FSSG) to survey the prevalence of GERD. In their report, a significant correlation between FSSG score and ≥ 3 cm of arm span – height difference was observed in elderly women. The present study also detected an association of HHL with the prevalence rate of GERD, which was diagnosed by endoscopic screening, in postmenopausal women. The significance of HHL as an independent determinant for GERD prevalence was ascertained by multiple logistic regression analysis adjusted for age and BMI. Furthermore, ROC curve analysis revealed the optimal cut-off value of HHL to discriminate the prevalence of GERD to be 5.35 cm. These newly uncovered findings suggest careful attention of elderly patients with ≥ 5 cm of HHL for the risk of GERD.

Height loss with aging has been associated with several co-morbidities, including respiratory disease, hypertension, vascular events, and GERD^4–6^. One cause for such disorders may be changes in fluid flow or airflow by a kyphotic deformity. In the present study, although hypertension and vascular events exhibited significant associations with HHL in quartile analysis, no remarkable relationships were seen in multiple logistic regression testing. In this analysis model, the impacts of age and BMI were very large; the OR of age and BMI for hypertension was 2.02 (95% CI 1.70–2.40) and 1.89 (95% CI 1.61–2.21), respectively, and the respective OR of age and BMI for vascular events was 2.13 (95% CI 1.67–2.72) and 1.44 (95% CI 1.20–1.72). This may partially explain the absent significant independence of HHL for the prevalence of these disorders.

Lastly, we detected a significantly higher presence of prevalent vertebral fracture and spinal osteoarthritis in GERD patients. Since the respective prevalence rate of vertebral fracture and spinal osteoarthritis was 1.7-fold and 1.3-fold higher than in non-GERD patients, the presence of GERD could represent a new criterion for detecting these vertebral disorders.

The present study had several limitations. First, since it employed arm span to estimate the patient's tallest height, there was possible measurement error due to contracture interfering with the extension of the subject's arms. Second, this investigation was a retrospective cross-sectional study of relatively small size that did not include male patients. Future prospective studies containing a large number of participants from a non-uniform population are required. Particularly, male patient data will be important for further understanding of the association between HHL and spinal osteoarthritis with GERD prevalence in more global cohorts.

In conclusion, this study demonstrated significant associations of HHL with prevalent vertebral fracture, spinal osteoarthritis, and GERD prevalence in postmenopausal women and the optimal HHL cut-off values for discriminating these factors. A significantly higher presence of vertebral disorders may be present in GERD patients as well. Better understanding of these relationships and proposed cut-off values will be useful for improving the diagnostic procedure, care management, and ultimately quality of life in elderly patients.

## Supplementary information

Supplementary Information

## Data Availability

The data analyzed or generated during the current study are available from the corresponding author on reasonable request.
