# Supplementary material for: Implications of historical height loss for prevalent vertebral fracture, spinal osteoarthritis, and gastroesophageal reflux disease
Source: Sci Rep. 2020 Nov 4;10:19036. doi: 10.1038/s41598-020-76074-6 (PMC7643061; doi:10.1038/s41598-020-76074-6)
Supplement: Supplementary file 1 — Supplementary Information [file 41598_2020_76074_MOESM1_ESM.docx]

**Supplementary Table 1.** ROC curve analysis of HHL to discriminate pVFx, sOA, GERD, and their combinations

|  | Optimal cut-off value (cm) | AUC (95% CI) |
| --- | --- | --- |
| pVFx | 4.95 | 0.740 (0.704–0.776) |
| sOA | 2.75 | 0.701 (0.667–0.735) |
| GERD | 5.35 | 0.692 (0.629–0.754) |
| pVFx + sOA | 5.65 | 0.774 (0.739–0.809) |
| pVFx + GERD | 5.95 | 0.792 (0.716–0.869) |
| sOA + GERD | 5.95 | 0.760 (0.701–0.820) |
| pVFx + sOA + GERD | 6.95 | 0.857 (0.805–0.909) |

ROC, receiver operating characteristic; HHL, historical height loss; pVFx, prevalent vertebral fracture; sOA, spinal osteoarthritis; GERD, gastroesophageal reflux disease; AUC, area under the curve; CI, confidence interval
